# Building capacity in the conservation of exceptional plant species

**DOI:** 10.1002/aps3.11498

**Published:** 2022-10-04

**Authors:** Megan Philpott, Valerie C. Pence, Emily E. D. Coffey

**Affiliations:** ^1^ Center for Conservation and Research of Endangered Wildlife Cincinnati Zoo & Botanical Garden, Cincinnati Ohio USA; ^2^ Southeastern Center for Conservation, Atlanta Botanical Garden Atlanta Georgia USA

The need for information on plant conservation techniques is increasing rapidly in the face of increased extinction risks to plants including habitat loss, unsustainable harvesting, and climate change (Antonelli et al., 2020). Ex situ techniques can be used as part of a toolkit to ensure that species are not lost before in situ conservation methods are able to address threats and that a stock of plants is available for future restoration efforts. The two primary methods used for conserving plants ex situ are living collections and seed banks. The reader is referred to several excellent reviews of current approaches to multi‐institutional living collections (Potter et al., 2017; Wood et al., 2020; Westwood et al., 2021). Seed banking has proved to be an efficient method for conserving large amounts of genetic material in a small space at a relatively low cost (Li and Pritchard, 2009), and the number of seed banking efforts worldwide is evidence of its success (Cochrane et al., 2007; Liu et al., 2018). However, while many species can be stored effectively in conventional seed banks (dried and frozen at −18°C or −20°C), some species cannot be seed banked using these methods and require alternative methods for their ex situ conservation: these are known as exceptional species.

A species may be considered exceptional for many reasons depending on the roadblock in the seed banking process. Pence et al. (2022a) define these roadblocks in terms of four exceptionality factors: EF1, species for which collecting seeds is difficult or impossible, or that have an exceptionally low seed number or viability; EF2, species with seeds that cannot survive drying sufficiently to allow survival through freezing; EF3, species with seeds that cannot survive being frozen long‐term at −20°C, the standard for conventional seed banking; and EF4, species with seeds that have deep or complex dormancies, making their recovery from banking difficult. Species with one or more exceptionality factors will need alternative conservation methods, either living collections or cryobanking. Successful cryobanking involves cryobiotechnologies and, for many species, in vitro propagation technologies, both of which must often be tailored to a particular species. In addition, data to determine a species' exceptional status are often lacking. The eight articles in this special issue, “Meeting the challenge of exceptional plant conservation: Technologies and approaches,” help fill those gaps by focusing on the following four areas.

## Identification of exceptional species using seed biology

The first crucial step of exceptional plant conservation involves identifying those species that are or are likely to be exceptional, by determining their desiccation tolerance, their longevity in dried, frozen storage, and/or any dormancies that might hinder germination. For many species, exceptional or not, seed germination requirements are poorly understood or unknown. Wolkis et al. (2022) address this issue with the exceptional species *Brighamia rockii* H. St. John by investigating dormancy and germination requirements. Using a systematic approach to uncovering germination requirements not only informs best practices for the conservation of the species but allows researchers to make inferences on the longevity of seed viability in both the seed bank and the wild. Experimental studies in seed biology can also lend insight into the ability of species to survive conventional seed banking long term. Seglias (2022) investigates the germination requirements of five alpine species and their likely tenure in storage using accelerated aging techniques. Alpine species in particular are often more likely to be short lived in seed banks than their lower‐elevation counterparts (Mondoni et al., 2011). By using accelerated aging, species can be targeted for alternative storage methods before their viability declines.

## Establishing, growing, and recovering plants from in vitro cultures

Once a plant is identified as being exceptional or potentially exceptional, the next step is to determine the most appropriate alternative conservation methods for that species. In vitro methods are required for preparing or recovering several of the tissue types that may be targeted for banking (Pence et al., 2020). For tissues that must be propagated in vitro, such as clonal shoots, initiation into tissue culture can be a major bottleneck to creating an in vitro collection. Winkeljohn et al. (2022) explore this issue in five species of *Quercus* L., using a known ethylene inhibitor in the initiation medium to improve initiation rates. Another challenge to establishing and growing in vitro tissues is their susceptibility to contamination by bacteria and fungi. Such contaminants may inhibit the initiation of a successful culture and can also remain as latent endophytes, proving lethal later in culture or in tissues recovering from the stress of cryopreservation. Volk et al. (2022) provide an overview of methods that can be used for controlling and eliminating contamination and use two case studies of *Citrus* L. to illustrate the process of identifying endophytes from these lines and the antibiotics that are most effective against them.

Many orchids are considered exceptional species due to the relatively short longevity of their seeds when frozen at −20°C (Merritt et al., 2014), and in vitro methods are routinely used for viability testing and germination of orchid seeds. Jolman et al. (2022) offer a comprehensive overview of the issues surrounding asymbiotic germination of orchid seeds, a process that allows practitioners to sidestep the difficulties of germinating species with their symbiotic mycorrhizal fungi. This work can give researchers a jumping‐off point for the development of in vitro germination protocols for new species in ex situ collections. In addition, Zale et al. (2022) investigate seed germination requirements of the widespread *Spiranthes* Rich. genus and develop reproducible in vitro methods for the propagation of the species. This work represents a major step forward in the conservation of *Spiranthes*, both in the production of plants for restoration projects and in its ex situ conservation.

## Cryopreservation and recovery of plant tissues

Cryopreservation is one of the most effective and reliable methods of long‐term storage for many exceptional species (Pence et al., 2020; Walters and Pence, 2021; Wang et al., 2021), but protocols and techniques are lacking for many species. Long‐term storage in liquid nitrogen provides many benefits, including reducing the space and labor needed to maintain living collections ex situ, extending the lifespan of many species in storage, and allowing for the banking of many different types of tissues besides seeds. Philpott et al. (2022) investigate the costs and resources needed for the cryopreservation of several exceptional species, information that can be used by practitioners considering the use of cryobiotechnologies in their management plans.

## Genetic issues in ex situ exceptional plant conservation

Any method of ex situ conservation must rely on a robust method of maintaining the genetic diversity of the species. This is particularly true with living collections, as they may be subject to genetic change through continuing cultivation and inbreeding. Foster et al. (2022) describe the importance of utilizing pedigree‐based management tools to identify and track diversity within and between collections residing at different botanical gardens, using case studies to provide guidance for targeting crosses and maintaining the genetic health of the collections through time.

## Steps toward the future

The challenge of exceptional plant conservation is considerable. Predictive models suggest that 8% of the total world flora have desiccation‐sensitive seeds (Wyse and Dickie, 2017), only one of the four potential causes for exceptionality. A recent analysis of the exceptional status of over 23,000 species identified 775 with enough information to be classified as exceptional (Pence et al., 2022b), a number far short of the tens of thousands of species predicted to be exceptional by modeling. Increased capacity in exceptional plant conservation will be needed to protect the most vulnerable species from extinction. Before undertaking the stewardship of an exceptional species, practitioners often have concerns about the associated costs and specific challenges. Philpott et al. (2022) provide an overview of the costs involved and approaches utilized by a variety of botanic gardens targeting exceptional species. While the magnitude of costs can be unpredictable due to the challenges of working with these species, labor consistently accounts for the largest proportion of costs, indicating the need to invest in practitioners themselves to improve conservation outcomes.

This special issue grew out of a number of collaborative exceptional plant conservation initiatives, including the collaborative grant approach detailed in Philpott et al. (2022) and the virtual Conservation of Exceptional Plants Symposium/Workshop hosted by the Cincinnati Zoo & Botanical Garden's Center for Conservation and Research of Endangered Wildlife (CREW) in October 2021. The goal of the symposium was to increase the number of resources available to practitioners around the world by hosting a free, online, workshop‐style conference, with the majority of conference materials made permanently accessible online through the Exceptional Plant Conservation Network (https://cincinnatizoo.org/conservation/crew/exceptional-plant-conservation-network/). A fresh infusion of resources, including expertise and funding, is necessary to build global capacity in the conservation of exceptional species, and increased awareness and education can be one means of achieving this. As more researchers study and develop conservation protocols for these vulnerable species and publish their results, collaboration, resources, and efficiency within the exceptional plant conservation community should increase. We hope that this special issue can contribute to that goal.

## AUTHOR CONTRIBUTIONS

M.P. prepared the first draft of the manuscript. M.P. and V.C.P. provided select article summaries. V.C.P. and E.E.D.C. provided reviewing and editing assistance. All authors approved the final version of the manuscript.

## References

[aps311498-bib-0001] Antonelli, A. , S. Hiscock , S. Lennon , M. Simmonds , R. J. Smith , and B. Young . 2020. Protecting and sustainably using the world's plants and fungi. Plants, People, Planet 2: 368–370. 10.1002/ppp3.10150

[aps311498-bib-0002] Cochrane, J. A. , A. D. Crawford , and L. T. Monks . 2007. The significance of ex situ seed conservation to reintroduction of threatened plants. Australian Journal of Botany 55: 356–361. 10.1071/BT06173

[aps311498-bib-0003] Foster, J. A. , S. K. Walsh , K. Havens , A. T. Kramer , and J. B. Fant . 2022. Supporting long‐term sustainability of ex situ collections using a pedigree‐based population management approach. Applications in Plant Sciences 10(5): e11491. 10.1002/aps3.11491 PMC9575128 36258785

[aps311498-bib-0004] Jolman, D. , M. I. Batalla , A. Hungerford , P. Norwood , N. Tait , and L. E. Wallace . 2022. The challenges of growing orchids from seeds for conservation: An assessment of asymbiotic techniques. Applications in Plant Sciences 10(5): e11496. 10.1002/aps3.11496 PMC9575117 36258786

[aps311498-bib-0005] Li, D.‐Z. , and H. W. Pritchard . 2009. The science and economics of ex situ plant conservation. Trends in Plant Science 14: 614–621. 10.1016/j.tplants.2009.09.005 19818672

[aps311498-bib-0006] Liu, U. , E. Breman , T. A. Cossu , and S. Kenney . 2018. The conservation value of germplasm stored at the Millennium Seed Bank, Royal Botanic Gardens, Kew, UK. Biodiversity and Conservation 27: 1347–1386. 10.1007/s10531-018-1497-y

[aps311498-bib-0007] Merritt, D. J. , F. R. Hay , N. D. Swarts , K. D. Sommerville , and K. W. Dixon . 2014. Ex situ conservation and cryopreservation of orchid germplasm. International Journal of Plant Sciences 175: 46–58. 10.1086/673370

[aps311498-bib-0008] Mondoni, A. , R. J. Probert , G. Rossi , E. Vegini , and F. R. Hay . 2011. Seeds of alpine plants are short lived: Implications for long‐term conservation. Annals of Botany 107: 171–179. 10.1093/aob/mcq222 21081585 PMC3002479

[aps311498-bib-0009] Pence, V. C. , D. Ballesteros , C. Walters , B. M. Reed , M. Philpott , K. W. Dixon , H. W. Pritchard , et al. 2020. Cryobiotechnologies: Tools for expanding long‐term ex situ conservation to all plant species. Biological Conservation 250: 108736. 10.1016/j.biocon.2020.108736

[aps311498-bib-0010] Pence, V. C. , A. Meyer , J. Linsky , J. Gratzfeld , H. W. Pritchard , M. Westwood , and E. B. Bruns . 2022a. Defining exceptional species—A conceptual framework to expand and advance ex situ conservation of plant diversity beyond conventional seed banking. Biological Conservation 266: 109440. 10.1016/j.biocon.2021.109440

[aps311498-bib-0011] Pence, V. C. , E. B. Bruns , A. Meyer , H. W. Pritchard , M. Westwood , J. Linsky , J. Gratzfeld , et al. 2022b. Gap analysis of exceptional species—Using a global list of exceptional plants to expand strategic ex situ conservation action beyond conventional seed banking. Biological Conservation 266: 109439. 10.1016/j.biocon.2021.109439

[aps311498-bib-0012] Philpott, M. , V. C. Pence , B. Bassüner , A. S. Clayton , E. E. D. Coffey , J. L. Downing , C. E. Edwards , et al. 2022. Harnessing the power of botanical gardens: Evaluating the costs and resources needed for exceptional plant conservation. Applications in Plant Sciences 10(5): e11495. 10.1002/aps3.11495 PMC9575053 36258792

[aps311498-bib-0013] Potter, K. M. , R. M. Jetton , A. Bower , D. F. Jacobs , G. Man , V. D. Hipkins , and M. Westwood . 2017. Banking on the future: Progress, challenges and opportunities for the genetic conservation of forest trees. New Forests 48: 153–180. 10.1007/s11056-017-9582-8

[aps311498-bib-0014] Seglias, A. E. 2022. Can alpine plant species “bank” on conservation?: Using artificial aging to understand seed longevity. Applications in Plant Sciences 10(5): e11493. 10.1002/aps3.11493 PMC9575086 36258790

[aps311498-bib-0015] Volk, G. M. , R. Bonnart , A. C. Araújo de Oliveira , and A. D. Henk . 2022. Minimizing the deleterious effects of endophytes in plant shoot tip cryopreservation. Applications in Plant Sciences 10(5): e11489. 10.1002/aps3.11489 PMC9575093 36258787

[aps311498-bib-0016] Walters, C. , and V. C. Pence . 2021. The unique role of seed banking and cryobiotechnologies in plant conservation. Plants, People, Planet 3(1): 83–91. 10.1002/ppp3.10121

[aps311498-bib-0017] Wang, M. R. , M. Lambardi , F. Engelmann , R. Pathirana , B. Panis , G. M. Volk , and Q. C. Wang . 2021. Advances in cryopreservation of in vitro‐derived propagules: Technologies and explant sources. Plant Cell Tissue and Organ Culture 144: 7–20. 10.1007/s11240-020-01770-0

[aps311498-bib-0018] Westwood, M. , N. Cavender , A. Meyer , and P. Smith . 2021. Botanic gardens solutions to the plant extinction crisis. Plants, People, Planet 3: 22–32. 10.1002/ppp3.10134

[aps311498-bib-0019] Winkeljohn, M. , V. C. Pence , and T. M. Culley . 2022. Improving culture initiation of mature oak shoots through use of silver thiosulfate. Applications in Plant Sciences 10(5): e11497. 10.1002/aps3.11497 PMC9575081 36258789

[aps311498-bib-0020] Wolkis, D. , C. C. Baskin , J. M. Baskin , and N. Rønsted . 2022. Seed dormancy and germination of the endangered exceptional Hawaiian lobelioid *Brighamia rockii* . Applications in Plant Sciences 10(5): e11492. 10.1002/aps3.11492 PMC9575077 36258791

[aps311498-bib-0021] Wood, J. , J. D. Ballou , T. Callicrate , J. B. Fant , M. P. Griffith , A. T. Kramer , R. C. Lacy , et al. 2020. Applying the zoo model to conservation of threatened exceptional plant species. Conservation Biology 34: 1416–1425. 10.1111/cobi.13503 32233087 PMC7754355

[aps311498-bib-0022] Wyse, S. V. , and J. B. Dickie . 2017. Predicting the global incidence of seed desiccation sensitivity. Journal of Ecology 105: 1082–1093. 10.1111/1365-2745.12725

[aps311498-bib-0023] Zale, P. J. , A. Clayton , J. Nix , and M. Taylor . 2022. Asymbiotic in vitro seed germination, in vitro seedling development, and ex vitro acclimatization of *Spiranthes* . Applications in Plant Sciences 10(5): e11494. 10.1002/aps3.11494 PMC9575095 36258788

